# *Lactobacillus johnsonii* N6.2 Phospholipids Induce T Cell Anergy upon Cognate Dendritic Cell Interactions

**DOI:** 10.3390/metabo15050284

**Published:** 2025-04-22

**Authors:** Alexandra E. Cuaycal, Monica F. Torrez Lamberti, Graciela L. Lorca, Claudio F. Gonzalez

**Affiliations:** Department of Microbiology and Cell Science, Genetics Institute, Institute of Food and Agricultural Sciences, University of Florida, Gainesville, FL 32610, USA; acuaycalbastidas@ufl.edu (A.E.C.); m.torrezlamberti@ufl.edu (M.F.T.L.); glorca@ufl.edu (G.L.L.)

**Keywords:** *Lactobacillus johnsonii* N6.2, microbial-derived lipids, phospholipid, dendritic cell, cellular immune response, immunomodulation, T cell response, anergy

## Abstract

**Background/Objectives**: *Lactobacillus johnsonii* N6.2 is a gut symbiont with probiotic properties. *L. johnsonii* N6.2 delayed the progression of type 1 diabetes (T1D) in diabetic-prone rats. The probiotic intake demonstrated immune cell modulation in healthy volunteers, leading to improved wellness and fewer reported symptoms like headaches and abdominal pain. These systemic immune-modulating benefits are attributed to *L. johnsonii* N6.2’s bioactive fractions, including extracellular vesicles (EVs) and purified phospholipids (PLs). We have previously shown that *L. johnsonii* N6.2 PLs modulate dendritic cell (DC) function towards a regulatory-like phenotype. Here, we further characterize the immune regulatory effects of *L. johnsonii* N6.2 PLs on adaptive immunity, specifically upon DC and T cell interactions. We hypothesized that PL-stimulated DCs suppress T cell-mediated responses to maintain tolerance in intra- and extra-intestinal sites. **Methods**: Bone marrow-derived dendritic cells (BMDCs) were generated from Sprague-Dawley rats and stimulated with *L. johnsonii* N6.2 PLs. Isogenic T cells were isolated from PBMCs obtained via terminal exsanguination. In vitro cellular assays, co-culture experiments, gene expression analysis by qRT-PCR, and flow cytometry assays were conducted to assess the immune regulatory effects of *L. johnsonii* N6.2 PLs. **Results**: The PL-stimulated BMDCs upregulated DC regulatory markers and exhibited an immature-like phenotype with reduced surface expression of maturation markers but increased surface migratory molecules (ICAM-1). These BMDCs presented immunosuppressive functions upon cognate T cell interactions and in the presence of TCR stimulation. Specifically, PL-stimulated BMCDs suppressed Th1 effector function and induced the expression of T cell anergy-related genes after co-culturing for 72 h. **Conclusions**: This study highlights the immune regulatory capacity of *L. johnsonii* N6.2’s bioactive components on adaptive immunity, specifically that of purified PLs on DC:T cell-mediated responses leading to immunosuppression. Our findings suggest that *L. johnsonii* N6.2-purified PLs play a role in regulating adaptive immunity, offering potential benefits for managing immune-related diseases like T1D.

## 1. Introduction

Oral administration of probiotic *L. johnsonii* N6.2 decreased T1D progression in diabetic-prone (BB-DP) rats [1]. In this animal model, a Th17 cell bias was observed in the MLNs of probiotic-fed BB-DP animals. This effect was directly associated with the *L. johnsonii* N6.2 protective effect, as diabetic-resistant (BB-DR) rats naturally showed a similar T cell polarization profile [2]. A phase 1 clinical trial using *L. johnsonii* N6.2 as a probiotic demonstrated important changes in the peripheral immune cell populations in healthy volunteers. The participants in the probiotic group reported fewer episodes of headaches, dizziness, abdominal pain, and indigestion [3]. Even though the benefits of the *L. johnsonii* N6.2 are evident, the bioactive components of *L. johnsonii* N6.2 mediating immune cell responses are less characterized.

Our group has also reported that *L. johnsonii* N6.2 releases extracellular vesicles (EVs) and that these vesicles activate AHR signaling to induce a tolerogenic profile in THP-1 macrophages. The anti-inflammatory activity of *L. johnsonii* N6.2 EVs reduces pancreatic beta-cell apoptosis in vitro [4]. These findings suggest that some of the beneficial properties of this probiotic may be linked to EV components. EVs mainly comprise lipids; therefore, we isolated and tested the immunogenic properties of different lipid species produced by *L. johnsonii* N6.2 on bone marrow-derived dendritic cells (BMDCs). Phospholipid (PL)-stimulated BMDCs maintained an immature-like phenotype with a significantly upregulated surface ICAM-1 [5].

Microbial-derived lipids have been studied for a long time in the context of lipid-containing PAMPs, PRR stimulation, and activation of innate immunity. Indeed, lipoproteins (LPPs) and lipopolysaccharide (LPS) have been thoroughly characterized given their importance in infection, pathogenesis, and inflammatory responses [6]. The suppression of innate immune responses by microbial lipids has also been reported in pathogenic bacteria [7,8,9,10,11]. Commensal-synthesized lipids play a major role in the tolerogenic process. Indeed, a correlation between *A. muciniphila* genome features and unique *A. muciniphila* lipid species in the cecum has recently been found [12]. Following this trend, recent studies have determined the immunoregulatory capacity of Gram-negative commensal-derived lipids, like α-GalCers synthesized by *Bacteroides* spp. [13,14,15,16,17], and, more recently, of phospholipids and ornithine lipids from *Akkermansia muciniphila* [12,18].

*Lactobacillus* strains have been shown to induce tolerogenic dendritic cells and regulatory T cells in vitro [19,20]. Moreover, their tolerogenic effects have also been demonstrated in vivo with animal models of disease [21,22,23]. Nonetheless, scientific reports on the involvement of commensal-derived lipids in modulating a host’s immunity are scarce.

*L. johnsonii* N6.2 purified PLs activated a non-canonical NF-κB pathway and induced a regulatory migratory-like transcriptional signature in BMDCs. This DC phenotype is important in the context of immune cell modulation. DCs can uptake, process, and present antigens (Ags) to T cells to regulate the adaptive immune response [24]. DCs can also present lipid antigens through their CD1 molecules, for the stimulation of lipid-reactive T cells and subsequent immune modulation [25]. Furthermore, DCs can induce tolerance to innocuous Ags, such as dietary- and commensal-derived Ags, under homeostatic conditions [26,27]. This tolerogenic activity is crucial to maintain tissue homeostasis and prevent autoimmunity [28,29].

To exert such activity, tolerogenic and steady-state DCs present an immature or semi-mature phenotypic profile. That profile is characterized by the low surface expression of costimulatory and MHC molecules, as well as anti-inflammatory cytokine production [30]. In the gut, DCs act in coordination with the gut microbiota, and such interactions depend on tailored immunoregulatory mechanisms that allow the host to recognize and tolerate commensals while fighting against pathogens [26]. The mechanism of how *L. johnsonii* N6.2 PL-stimulated dendritic cells exert their immune cell modulation remains to be determined.

In this context, the primary objective of this research was to assess the T cell-modulating potential of *L. johnsonii* N6.2 PL-stimulated dendritic cells. We hypothesized that PL-pulsed dendritic cells suppress T cell-mediated responses to maintain tolerogenic conditions in the gut and other extra-intestinal sites, as previously reported [1,4,31]. Our results suggest that *L. johnsonii* N6.2 PL-stimulated BMDCs maintain an immature-like phenotype with migratory capacities, suggesting that PL-pulsed BMDCs may migrate to other body sites to exert their suppressive functions. They suppressed the Th1 effector function upon interactions with cognate T cells, triggering the expression of T cell anergy-related genes.

## 2. Materials and Methods

### 2.1. Animals

The experiments described here were performed with primary cell lines generated from 8–12-week-old Sprague-Dawley (SD) rats. The animals were purchased from Charles River Laboratories (Wilmington, MA, USA) and housed under a 12 h light/dark cycle and controlled climate as prescribed by the Association for Assessment and Accreditation of Laboratory Animal Care. The animal protocol and animal procedures were approved by the University of Florida Animal Care and Use Committee.

### 2.2. Generation of BMDCs and Stimulation with L. johnsonii N6.2 Extracted Lipids

*L. johnsonii* N6.2 total lipids (TL) and phospholipids were isolated as previously described [5]. In short, *L. johnsonii* N6.2 cells from a 24 h growth culture were harvested by centrifugation, washed twice with 1% (*w*/*v*) NaCl, frozen at −80 °C overnight and freeze-dried for 24 h. Total lipids were extracted using a modified Bligh and Dyer method using a final chloroform/methanol/water ratio of 1:2:0.8 (*v*/*v*/*v*) and dried under a N_2_ stream. The TLs were further fractionated by column chromatography using a modified Frostegård method [32]. The TLs were resuspended in chloroform:acetic acid (100:1 *v*/*v*) and loaded to a column (3.2 × 45.7 cm) of oven-activated (125 °C) silica gel 60 (0.063–0.200 mm, MilliporeSigma, Burlington, MA, USA) that was conditioned with the same solvent mixture. A simple lipid fraction (SL, including fatty acids) was eluted with chloroform:acetic acid (100:1 *v*/*v*), followed by a glycolipid (GL) fraction eluted with 100% acetone and a phospholipid (PL) fraction eluted with 100% methanol.

The BMDCs were generated from bone marrow (BM) progenitors from the femurs of SD rats. The BM cells were resuspended in RPMI-1640 complete medium (CM). The CM was prepared with RPMI-1640 supplemented with 10% heat-inactivated fetal bovine serum (FBS, Sigma-Aldrich, Inc., St. Louis, MO, USA), 2 mM L-glutamine (as GlutaMAX^TM^), 50 µM 2-mercaptoethanol (Sigma-Aldrich, Inc., St. Louis, MO, USA), 100 units/mL of penicillin, and 100 µg/mL streptomycin (all components were from Gibco, TermoFisher, Waltham, MA, USA, unless otherwise detailed). To generate BMDCs, the CM was supplemented with 20 ng/mL of rat GM-CSF and 200 ng/mL of human FLT3-L (both were from R&D Systems, Minneapolis, MN, USA). The BMDCs were cultured for 7 days at 37 °C and 5% CO_2_ in untreated T-75 flasks (Thermo Scientific, TermoFisher, Waltham, MA, USA). The culture medium was changed every 2–3 days. The purity of the non-adherent BMDCs after seven days of culture was confirmed by flow cytometry with anti-rat CD11b/c antibody (Clone OX-42, BioLegend, San Diego, CA, USA) and was routinely > 90%.

The culture stimulations were as follows: 3 × 10^4^ BMDCs were stimulated with either *L. johnsonii* N6.2 PLs at 5 µg/mL or the vehicle control in 24-well untreated plates (Thermo Scientific, TermoFisher, MA, USA) for 24 h in CM. After the incubation period, the PL-stimulated BMDCs were washed and resuspended in RPMI-1460 supplemented with 2.5% FBS substitute (Biolegend, San Diego, CA USA), 2 mM L-glutamine (as GlutaMAX^TM^), 50 µM 2-mercaptoethanol (Sigma-Aldrich, Inc., St. Louis, MO, USA), 100 units/mL of penicillin, 100 µg/mL streptomycin (T-cell-CM). All components were from Gibco (TermoFisher, MA, USA) unless otherwise indicated. The PL-stimulated BMDCs were used for co-culture experiments and analyzed by flow cytometry or lysed for total RNA isolation as described below.

### 2.3. Isolation and Maintenance of Homeostatic Peripheral T Cells

Terminal exsanguination was performed via cardiac puncture under a deep level of anesthesia to collect total blood from the SD rats following standard animal procedures [33]. The blood was collected into heparin collection tubes and processed immediately. Peripheral blood mononuclear cells (PBMCs) were isolated by density gradient centrifugation with Ficol Paque [34]. Peripheral T cells were isolated by magnetic activated cell sorting (MACS) with rat pan T cell MicroBeads (Miltenyi Biotec, San Jose, CA, USA). Enriched T cells were maintained in culture with T-cell-CM medium supplemented with 1 ng/mL of IL-7 and 10 ng/mL of IL-2 [35,36] (both were from R&D Systems, MN, USA). Alternatively, enriched T cells were resuspended in freezing media (10% DMSO in FBS Sigma-Aldrich, Inc., St. Louis, MO, USA) and frozen in a slow-freezing container at −80 °C until use. The purity and phenotype of enriched and maintained/cryopreserved T cells were followed by flow cytometry. The purity of T cell isolation was consistently over 95%, as determined by anti-CD3 staining followed by flow cytometry. For co-culture experiments, T cells were washed and resuspended in complete T cell medium without addition of cytokines.

### 2.4. Co-Culture Experiments

**Figure 1:**
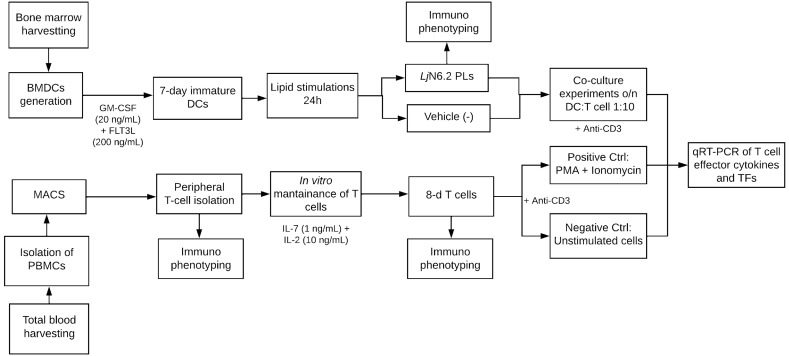
Schematic representation of co-culture experiments performed.

PL-stimulated BMDCs (3 × 10^4^) were co-cultured with peripheral T cells (3 × 10^5^), at a ratio of 1:10 in 24-well plates for 18 h. A schematic representation of the co-culture experiments performed is displayed in Figure 1. Subsequently, the cells were harvested, and total RNA was isolated for gene expression analysis of T effector Th1, Th2, Th17, and Teg cytokines *Ifng*, *Il4*, *Il17a*, *Il10* and transcription factors (TFs) *Tbx21*, *Gata3*, *Rorc*, and *Foxp3*, respectively, as well as T cell anergy-related genes. Co-culture experiments with cryopreserved T cells were performed as described above.

### 2.5. RT-qPCR Analyses

Total RNA from the cells was isolated with the RNeasy mini kit (Qiagen, Germantown, MD, USA), following the manufacturer’s protocol. Contaminant DNA was digested with the TURBO DNA-freeTM kit (Invitrogen, TermoFisher, MA, USA). cDNA was generated with the iScriptTM cDNA synthesis kit (Bio-Rad, Hercules, CA, USA), and quantitative (q)PCR was performed using the PowerUp™ SYBR™ Green Master Mix (Applied Biosystems, TermoFisher, MA, USA) and a QuantStudio 6 machine (Applied Biosystems, TermoFisher, MA, USA). *Gapdh* was used as an internal control for BMDC measurements and β-actin in the case of T cell experiments. The primers used were designed to specifically bind rat cDNA. The primers’ sequences are shown in Table 1.

### 2.6. Flow Cytometry Analysis

The BMDCs and T cell phenotype were characterized with a panel of cell surface and intracellular (for T cells) markers with mouse anti-rat antibodies (Abs) conjugated to different fluorophores/biotin. The centrifugation steps were performed at 300× *g*, for 5 min. The Ab cocktail for the BMDC phenotype was prepared with BD Horizon™ Brilliant Stain Buffer (BD Biosciences, Franklin Lakes, NJ, USA) and each corresponding Ab: PE/Cy7-OX-42 Ab (anti-CD11b/c, BioLegend, CA, USA), PerCP-OX-6 Ab (anti-MHC-II, BD Biosciences, NJ, USA), FITC-24F Ab (anti-CD86, BioLegend, CA, USA), biotin-3H5 Ab (anti-CD80, Thermo Fisher Scientific, Waltham, MA, USA), BV711-1A29 Ab (anti-ICAM-1, BD Biosciences, NJ, USA), APC-HM40-3 Ab (anti-CD40, Thermo Fisher Scientific, MA, USA), and BV421-WTH1 Ab (anti-CD1d, BD Biosciences, NJ, USA). For the T cell panel, the cells were stained with PerCP anti- αβ T-Cell Receptor (Clone R73, BD Biosciences, NJ, USA), BV421 anti-CD161a (BD Biosciences, NJ, USA), BV711 anti-CD4 (BD Biosciences, NJ, USA), BV510 anti-CD8b (BD Biosciences, NJ, USA), APC anti-CD3 (BioLegend, CA, USA), BUV 737 anti-CD25 (BD Biosciences, NJ, USA), PE anti-PLZF (eBioscience, Thermo Fisher Scientific, MA, USA), Alexa Fluor 488 anti-mouse/human/rat FOXP3 (BioLegend, CA, USA).

Both the BMDCs and the T cells were spun down and washed twice with PBS prior to staining. Viability was assessed with the eFluorTM 780 fixable viability dye (FVD) (Invitrogen, MA, USA) for 30 min at 4 °C. After FVD incubation, the cells were washed two times with staining buffer (BioLegend, CA, USA) and then blocked with mouse anti-rat CD32 Ab for 10 min at room temperature (RT). After 10 min, the Ab cocktail was added, and the cells were incubated for an additional 30 min at RT. The cells were washed again two times with staining buffer and fixed with the Cyto-Fast™ Fix/Perm Buffer Set (BioLegend, CA, USA). The samples were run in an Aurora 5 (Cytek) instrument, and the data were analyzed with the FCS Express 7 Flow software.

### 2.7. Statistical Analysis

All statistical analyses were performed in R Studio (version 2022.7.1.554). Statistically significant gene expression between treatments in co-culture experiments was determined with a one-way ANOVA followed by a Tukey post hoc test with the log2 values. Protein expression by flow cytometry was analyzed as median fluorescence intensity (MFI), and differences among treatments were determined with a one-way ANOVA and a Tukey test. Statistical significance was defined with a *p* value < 0.05.

## 3. Results

### 3.1. L. johnsonii N6.2 Phospholipids Reduce the Surface Expression of Maturation Markers in BMDCs

**Figure 2:**
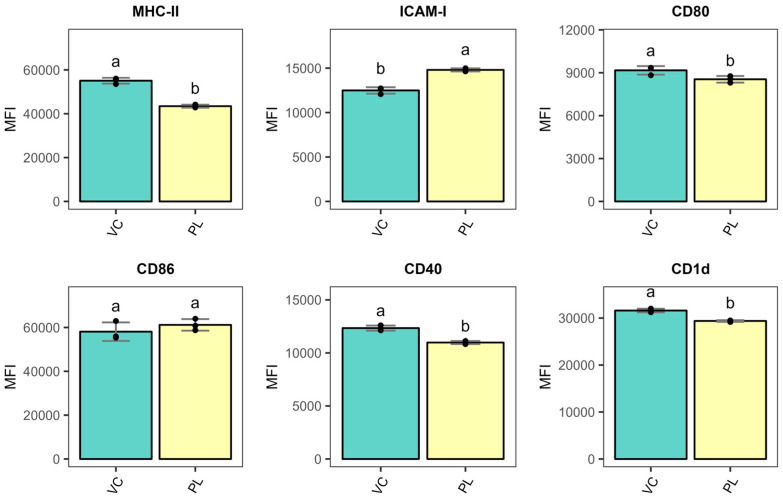
Immunophenotyping of BMDCs after 24 h stimulations with *L. johnsonii* N6.2 phospholipids. The results present median fluorescence intensity (MFI) values. Different letters denote statistical significance (*p*-value < 0.05). VC: Vehicle control; PLs: Phospholipids. MHC-II: Major Histocompatibility Complex II; ICAM-1: Intercellular Adhesion Molecule 1; CD: Cluster of differentiation.

**Figure 3:**
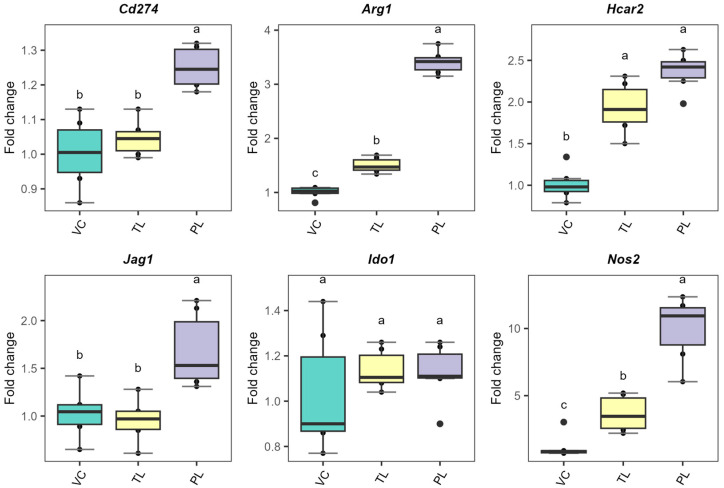
Gene expression analysis of dendritic cell tolerogenic markers after 24 h of incubation with *L. johnsonii* N6.2 purified lipids. VC: Vehicle control, TL: total lipids, PL: phospholipids. *Cd274*: Cluster of differentiation 274 (encoding PD-L1 molecule); *Arg1*: Arginase 1; *Hcar2*: Hydroxycarboxylic acid receptor 2; *Jag1*: Jagged 1; *Ido1*: Indoleamine-pyrrole 2,3-dioxygenase; *Nos2*: Nitric oxide synthase 2. Different letters denote statistical significance (*p*-value < 0.05).

The BMDCs were isolated according to the experimental design depicted in Figure 1. First, the BMDCs were stimulated in the presence or absence of total lipids or PLs to recapitulate the phenotype previously reported. The immunophenotyping after the incubation period confirmed that PL-stimulated BMDCs maintain an immature-like phenotype (Figure 2), similar to the profile obtained with total lipids [5]. We observed significantly reduced surface MHC-II, CD80, CD40, and CD1d upon stimulations for 24 h. Surface ICAM-1 was significantly increased in agreement with our previous report [5]. These results support the induction of a migratory-like phenotype on immature-like BMDCs. We also investigated the gene expression of tolerogenic markers after incubating the BMDCs with *L. johnsonii* N6.2 TL or PL fractions. Figure 3 shows the comparative results obtained with PL and TL. We observed a significant upregulation of arginase1 (Arg1), PD-PL (Cd274), GPR109a (Hcar2), and Jagged-1 (Jag-1) with PLs. Interestingly, we also observed the upregulation of inducible nitric oxide synthase (Nos2), suggesting that the lipid stimulation induced a metabolic reprogramming in the BMDCs towards a tolerogenic, immature-like phenotype.

### 3.2. Expansion of CD8+ CD161+ Memory T Cells from Endogenous Peripheral T Cells

Peripheral T cells were isolated from PBMCs by positive selection with MACS and kept in culture with 1 ng/mL of IL-7 and 10 ng/mL of IL-2. These cytokines have been previously shown to promote naive T cell survival and size maintenance [35]. After 8 days of culture, the T cells were harvested, washed, and utilized for co-culture experiments with PL-stimulated BMDCs. The phenotype of the peripheral T cells was evaluated after isolation from fresh PBMCs and after maintenance in vitro. Cell viability was consistently over 85–90% for the fresh and cultured T cells (Figure S1). The culture conditions induced upregulation of surface CD25 in both CD4+ and CD8+ T cells in concordance with the IL-2 supplementation. Nonetheless, the frequency of CD8+ T cells increased from about 20% to 50% while the CD4+ T cells were reduced. Interestingly, we also observed an expansion of CD161+ T cells (Figure S2). Gating for CD8+ T cells revealed the presence of CD161+ cells with low–high expression. This phenotype is consistent with the expansion of CD8+ CD161+ memory T cells (Figure S3A). Furthermore, FOXP3 staining showed the presence of a small percentage of FOXP3+ CD8+ T cells within this population. In addition, there were no CD4+ FOXP3+ T cells after the incubation period (Figure S3B).

### 3.3. PL-Stimulated BMDCs Facilitate the Expression of Anergy-Related Genes in T Cells

**Figure 4:**
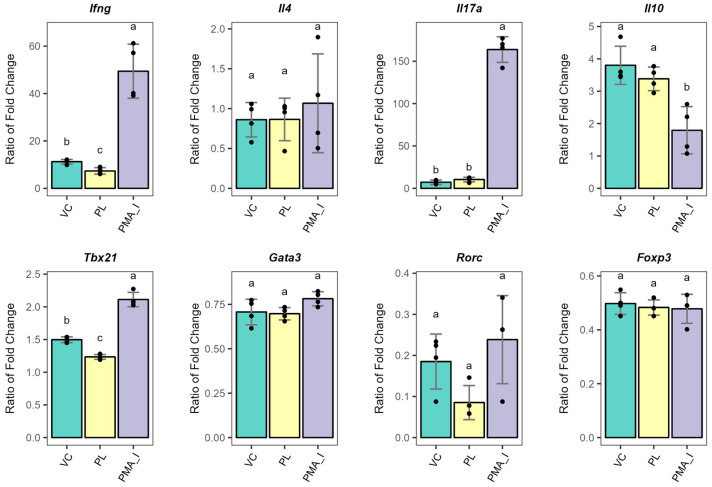
Gene expression analysis of T effector function cytokines and transcription factors upon co-culture with PL-stimulated BMDC and TCR stimulation for 18 h. Gene expression data were normalized to *Actb* and results for treatments are presented relative to that of unstimulated T cells. As a positive control, T cells alone were treated with PMA and Ionomycin to downstream TCR signaling. Different letter labels denote statistically significant changes (*p*-value < 0.05). VC: Vehicle control; PL: Phospholipids, PMA_I: PMA/Ionomycin. *Ifng*: Interferon gamma; *Il*: interleukin; *Tbx21*: T-box transcription factor; *Gata3*: GATA-binding factor 3; *Rorc*: Retinoic acid-related orphan receptor C; *Foxp3*: forkhead box P3.

To evaluate the function of PL-stimulated BMDCs on T cell responses, we conducted a co-culture experiment with peripheral T cells in the presence of TCR stimulation. A schematic representation of the co-culture experiments performed is displayed in Figure 1. The T cell population tested represented endogenous polyclonal CD4+ and CD8+ T cells. To this end, the BMDCs were stimulated with *L. johnsonii* N6.2 phospholipids for 24 h, washed, and further incubated with cultured T cells in a ratio of 1:10 for 18 h. Co-culture experiments were carried out in the presence of anti-CD3 to provide TCR stimulation. As a positive control of T cell function, T cells alone were treated with PMA and Ionomycin to induce cytokine expression (PMA_I treatment). Unstimulated T cells alone were also cultured in similar conditions. Gene expression analysis of T cell effector cytokines *Ifng, Il4, Il17a*, and *Il10*, along with the transcription factors *Tbx21*, *Gata3*, *Rorc*, and *Foxp3* for the Th1, 2, 17, and Treg subsets, respectively, was determined by qRT-PCR (Figure 4). The data are presented relative to the gene expression levels of the unstimulated T cells. Remarkably, the *L. johnsonii* N6.2 PLs specifically lowered the expression levels of *Ifng* and *Tbx21*, suggesting that PL-stimulated BMDCs may regulate Th1 effector functions in vitro. We observed similar results with PL-stimulated BMDCs co-cultured with cryopreserved T cells (Figure S4). Thus, the downregulation of Th1 effector genes was specific to the PL stimulation.

**Figure 5:**
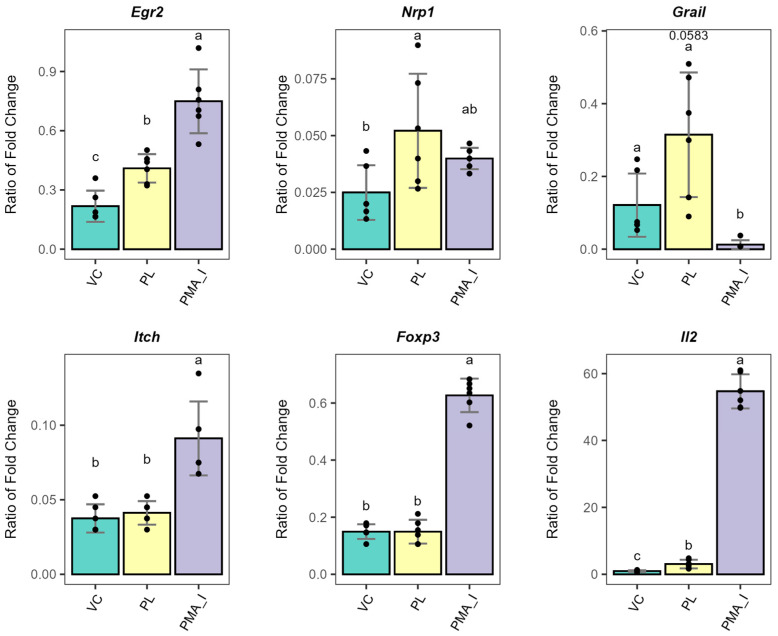
Gene expression levels of T cell anergy markers in cryopreserved T cells upon co-culture with PL-stimulated BMDC and TCR stimulation for 72 h. Gene expression data were normalized to *Actb*, and results for treatments are presented relative to that of unstimulated T cells. As a positive control, T cells alone were treated with PMA and Ionomycin to induce downstream TCR signaling (PMA_I). Different letter labels denote statistically significant changes (*p*-value < 0.05). VC: Vehicle control; PL: Phospholipids; PMA_I: PMA/Ionomycin. *Egr2*: Early growth response protein 2; *Nrp1*: Neuropilin 1; *Grail*: Gene related to anergy in lymphocytes; *Itch*: Itchy E3 Ubiquitin Protein Ligase; *Foxp3*: forkhead box P3; *Il*: interleukin.

Next, the mRNA expression levels of T cell regulatory and/or anergy induction markers in the experiments with cryopreserved T cells after 72 h of co-culture were evaluated (Figure 5). Remarkably, the upregulation of *Egr2* and *Nrp1* was observed. *Egr2* expression is upregulated and maintained long-term (2–5 days) in anergic T cells [37], whereas the expression of *Nrp1* is observed after anergy has been established [38]. In addition, *Nrp1*-expressing cells can transition into Tregs without *Foxp3* expression, which is consistent with the gene expression results [38,39]. Furthermore, *Egr2* upregulation was observed without co-stimulation, which is consistent with the PL-stimulated BMDC phenotype [40]. In contrast, we did not observe significant upregulation of *Grail* and *Itch*, additional markers of anergy induction. Nonetheless, *Grail* was trending towards higher expression (*p*-value = 0.058). *Grail* expression was previously reported to be upregulated rapidly and observed at 4 h post-stimulation [41]. Consequently, the gene expression profile observed here after 72 h post-stimulation is consistent with established T cell anergy. Interestingly, a slight upregulation of the cytokine IL-2 (*Il2*) was observed when the T cells were co-cultured with PL-stimulated BMDCs, suggesting the sustained T cell anergic profile. Overall, our results demonstrate suppression of Th1 effector function and upregulation of anergy markers, suggesting that PL-stimulated BMDCs suppress T cell responses through anergy induction.

## 4. Discussion

Recent efforts have demonstrated that the gut microbial lipidome is unique and structurally different from host-derived lipids [12,42]. Strikingly, these highly diverse commensal-derived lipids are increasingly being recognized as bioactive modulators of host immunity [5,14,17,18,43]. The activation of lipid-reactive iNKT cells by *Bacteroides*-derived sphingolipids is well defined [13,14,17]. Conversely, little is known about the T cell-stimulating potential of commensal-derived phospholipids. A recent study demonstrated that *E. coli*-derived cardiolipin (CL), phosphatidylglycerol (PG), phosphatidylethanolamine (PE), and total polar lipids modulate homeostasis and function of hepatic γδ-17 T cells in a CD1d-dependent manner [44]. In addition, PG derived from the skin colonizers and opportunistic pathogens *S. aureus* and *S. epidermidis* were found to activate CD1a-dependent CD4+ T cell responses. Nonetheless, those CD1a-restricted T cells were found to be a heterogeneous subset expressing Th2-, Treg- and cytotoxic-related genes [45].

The gut is the most immunologically active body site. Dietary- and commensal-derived Ags tightly regulate intestinal immunity. BMDCs play a crucial role in such regulations, as they can prime and polarize distinct T cell responses. Indeed, T cell effector programs are either inflammatory in defense against pathogens or tolerizing in response to innocuous Ags [26,27]. DCs exert such divergent outcomes due to their heterogeneous functional and maturation states [46,47]. Importantly, DCs also maintain tissue homeostasis in response to self-Ags by suppressing T cell activation. This control is critical to prevent autoimmune responses to endogenous Ags under proinflammatory conditions. In that context, steady-state DCs induce T cell clonal anergy, deletion, and conversion to peripheral Tregs in the absence of pro-immunogenic signals [28]. Such suppressive mechanisms partially depend on low-costimulatory signals provided by the Ag-presenting DC during TCR engagement [30].

Our results demonstrated that *L. johnsonii* N6.2 PL stimulations in BMDCs induce significant downregulation of surface costimulatory molecules (CD80 and CD40) along with the peptide-presenting MHC-II and lipid-presenting CD1d. Those BMDCs also significantly upregulated surface ICAM-1 (Figure 2). Consistent with that, upregulation of DC tolerogenic markers was observed (Figure 3). These results further support the reported maintained immature-like state on PL-stimulated BMDCs [5]. ICAM-1 is a cell-adhesion molecule important for DC homing to lymph nodes (LNs) [48]. Once there, T cell migration within the LN is ICAM-1-dependent [49]. In addition, this molecule also sustains stable T cell–DC contact interactions, enhances Ag discrimination and tunes T cell signaling [50,51,52]. Hence, surface expression of ICAM-1 is critical for DC-T cell interactions. The increased surface ICAM-1 in the DC phenotype observed here is implicated in immunosuppression, as clonal anergy of T cells has previously been reported upon cognate stimulation in the presence of MHC-II and ICAM-1 [53].

A recent report showed that phosphatidylserine (PS) autoantigen-containing liposomes reduce the maturation of human DCs from healthy and T1D subjects. Indeed, those PS liposome-stimulated DCs exhibited similar HLA-DR, CD40, CD86, and ICAM-1 levels than immature DCs. Interestingly, PS liposome-stimulated DCs significantly decreased autologous CD8+ proliferation compared to immature DCs. However, the effect was reverted when cytokine-induced matured DCs were used [54]. In addition, the control of DC maturation by microbial-derived lipids has previously been reported in *Mycobacterium tuberculosis*. Its cell surface glycolipids have been shown to bind DC-SIGN and suppress the upregulation of surface costimulatory molecules. This regulation also inhibited TLR-induced maturation and promoted *M. tuberculosis* evasion of innate immunity [8]. A similar phenotype is induced upon DC stimulation with *Fasciola hepatica* tegumental Ags [55]. Remarkably, these helminth-derived Ags were shown to induce DC-mediated anergy of T cells [56]. Importantly, helminth-derived immunoregulatory components have been suggested as a potential therapy in autoimmune diseases because of their immunosuppressive activity [57].

The suppressive functions of PL-stimulated BMDCs were evaluated on peripheral polyclonal CD4+ and CD8+ T cells. In this report, we first optimized the isolation and expansion of peripheral T cells from rats; the supplementation of IL-7 and IL-2 in the culture medium induced the expansion of CD8+ CD161+ “memory” T cells. Interestingly, IL-7 and IL-2 have previously been shown to maintain naïve T cell survival and size upon in vitro culture [35,36]. IL-7 also maintains survival and homeostatic intermittent proliferation of memory T cells [58]. In agreement with previous reports, our culture conditions not only maintained the survival of CD4+ and CD8+ populations (Figure S1) but also promoted the proliferation of CD8+ memory T cells (Figures S2 and S3A). Furthermore, the expansion of CD4+ FOXP3+ T cells (Figure S3B) upon IL-2 supplementations was not observed.

CD161+ CD8+ T cells have been shown to display Th1/Th17 functions. These memory T cells not only circulate in blood but also reside in mucosal sites. Upon mitogen stimulation (PMA + inonomycin + brefeldin A) and costimulatory signals, the cells secrete IFNγ and IL-17 [59]. Certainly, we observed a significant induction of *Ifng* and *Il17a* concomitant with significantly reduced *Il10* after PMA and ionomycin stimulations of T cells alone (Figure 4). This treatment constituted our positive control and demonstrated the functional state of cultured T cells. Nonetheless, our analysis could not discriminate between CD4+ and CD8+ T cell responses.

Upon interactions with T cells, tolerogenic and/or steady-state BMDCs induce clonal T cell anergy, deletion, or induction of regulatory T cells (Treg). Induction of Tregs is mediated by the immature-like phenotype (absence of co-stimulation) and secretion of IL-10 and TGFβ. On the other hand, T cell anergy and deletion are induced by the expression of inhibitory receptors (PDL-1 on DC or CTLA-4 on T cells) and apoptosis-inducing molecules (Fas/FasL or TRAIL/TRAILR interactions), respectively (extensively reviewed in [29]). The different immunosuppressive mechanisms rely on the DC subset and location of BMDCs exerting such regulation. Remarkably, the co-incubation of T cells with PL-stimulated BMDCs significantly reduced *Ifng* and *Tbx21* gene expression levels (as opposed to vehicle control-treated cells); this is consistent with *L. johnsonii* N6.2 PL-mediated regulation of Th1 effector functions. This regulatory profile has previously been shown for commensal *S. aureus*-derived LPPs. Indeed, lower levels of IFNγ were secreted by peripheral CD4+ T cells upon co-incubation with LPP-stimulated moBMDCs. Interestingly, the lipid moiety of LPPs from commensal and non-commensal staphylococci differentially regulate such responses [60].

T cells co-cultured with PL-stimulated BMDCs displayed lower levels of *Il2* and *Infg* gene expression, associated with higher gene expression levels of *Erg2* and *Nrp1* (Figure 5). The combination of these markers’ expression patterns is consistent with an anergy-like T cell’s dysfunctional state [39]. Notably, probiotics like *Lactobacillus casei* have also been associated with the ability to trigger T cell anergy [61]. Thus, our results suggest that *L. johnsonii* phospholipids could mediate peripheral tolerance mechanisms in adaptive immunity. This immune suppression may contribute to maintaining immune homeostasis and lowering the autoimmunity rate in BB-DP animals [1].

Therefore, T cell anergy induction in the gut carries several important implications for immune regulation. Beyond promoting tolerance to commensal bacteria and preventing autoimmunity, it also contributes to maintaining a balanced response to pathogens, increasing susceptibility to infections. Conversely, insufficient anergy and immunosuppression can lead to inappropriate immune activation and sustained inflammation. Thus, a gut microbial imbalance can disrupt immune homeostasis and contribute to gut immune dysfunction, which leads to local and systemic inflammatory conditions [62,63].

We have previously reported the immune-stimulating capacity of different *L. johnsonii* N6.2 bioactive fractions, including whole cells, EVs, purified lipids, and protein effectors [2,3,4,5,64,65,66]. Indeed, our pre-clinical studies demonstrated the upregulation of a Th17-related profile in the mesenteric lymph nodes of BB-DP rats that were orally administered the probiotic, including upregulation of IL-6 cytokine [2]. Similarly, we have observed that purified lipids and exopolysaccharides from *L. johnsonii* N6.2 induce this cytokine along with IL-10 in DCs [5]. Furthermore, EVs produced from this probiotic bacterium induce an anti-inflammatory profile in Thp1 macrophages [4] and an RNA-sensing-like response in human beta cell lines (Betalox5). In line with that, EVs reduced viral infection concomitant with the activation of the 2′,5′-oligoadenylate synthetase (OAS) pathway in an animal model of norovirus infection [65,66]. Strikingly, the anti-viral protective response was specific to the SH3b2 domain of the Sdp protein that is enriched in *L. johnsonii* N6.2 EVs but not *L. johnsonii* N6.2-generated liposomes.

## 5. Conclusions

Here, we further demonstrate the immune-stimulating capacity of *L. johnsonii* N6.2 bioactive components on the adaptive component of the immune response, specifically the immunosuppressive capacity of PL-stimulated BMDCs upon interaction with T cells. Our results described here, as well as our previous studies, highlight that the immune-stimulating capacity of *L. johnsonii* N6.2 is bioactive fraction-specific and cell type-specific, including modulation of both innate and adaptive immune cell activity with systemic effects. Our results have significant implications for gut commensal-mediated regulation of host immunity. Specifically, under dysbiosis conditions, which trigger both acute and chronic inflammation, there is an increased risk of developing intra- and extra-intestinal diseases, including type 1 diabetes (T1D). The results reported here provide a mechanistic framework for our previous reports, in which *L. johnsonii* N6.2 supplementation had a positive effect on health [1,3,31,67]. Furthermore, our results suggest that *L. johnsonii* N6.2-derived phospholipids may regulate the host’s adaptive immunity and restore health. To the best of our knowledge, such regulation and immunosuppression have not been reported for gut commensal-derived lipids.

## Figures and Tables

**Table 1:** Primers used for RT-qPCR analysis.

Gene	Forward Sequence (5′-3′)	Reverse Sequence (5′-3′)
*Cd274*	CAGTCTCCTCGCCTACAGGT	GCTGTGATGGTAAATGCCGC
*Arg1*	CAGTATTCACCCCGGCTACG	AGTCCTGAAAGTAGCCCTGTCT
*Hcar2*	ACATGATGACCCGAAACGGC	AGCAGAACAGGATGATGCCC
*Jag1*	ATGCCTCCTGTCGGGATTTG	CAGTGACCCCCATTCAAGCA
*Ido1* *	AGCACTGGAGAAGGCACTGT	ACGTGGAAAAAGGTGTCTGG
*Nos2* **	CTCACTGTGGCTGTGGTCACCTA	GGGTCTTCGGGCTTCAGGTTA
*Actb*	ACACCCGCCACCAGTTCG	CACGATGGAGGGGAAGACGG
*Ifng*	GTGTCATCGAATCGCACCTGA	GATCTGTGGGTTGTTCACCTCG
*Il4*	TTACGGCAACAAGGAACACCA	CACCGAGAACCCCAGACTTG
*Il17a*	CCTGGACTCTGAGCCGCAAT	ACTTCCCCTCAGCGTTGACA
*Il10*	CTGGTAGAAGTGATGCCCCA	GGAGAAATCGATGACAGCGT
*Tbx21*	GAGCCCACGAGCCATTACAG	CGTATAAGCGGTTCCCTGGC
*Gata3*	ATGGTCAAGGCAACCACGTC	CATACCTGGCTCCCGTGGTG
*Rorc*	GTACGTGGTGGAGTTCGCC	CGACTTCCATTGCTCCTGCTT
*Foxp3*	ACCCAGGAAAGACAGCAACCTT	TTCTCACAACCCGGCCACTT
*Egr2*	CTGCCTGACAGCCTCTACCC	CAATGTTGATCATGCCATCTCCAG
*Nrp1*	TGGGCTGTGAAGTAGAAGTGCC	CTCCTGTGAGCTGGAAGTCATC
*Grail*	AGCTCTGGGAATTGAGGTGGA	GTTGTCCTCTTCGTGGGGAG
*Itch*	TCGCTGTAGTCGGGGCT	GTGAAATGCATGTTACCGGGAC
*Il2*	TGTTGCTGGACTTACAGGTGC	ATGTTTCAATTCTGTGGCCTGCTT

*: Primers sourced from [31]. **: Primers sourced from [1]. All other primers were designed for this work.

## Data Availability

The original contributions presented in this study are included in the article/Supplementary Material. Further inquiries can be directed at the corresponding author.

## Supplementary Materials

The following supporting information can be downloaded at https://www.mdpi.com/article/10.3390/metabo15050284/s1, Figure S1: Purity and CD3 staining of fresh and cultured T cells; Figure S2: Immunophenotyping of peripheral T cells at the start compared to 8 days of culture; Figure S3: Immunophenotyping of peripheral CD8+ T cells; Figure S4: Gene expression levels of Th1 effector-related genes in cryopreserved T cells upon co-culture with PL-stimulated BMDCs and TCR stimulation for 18 h.

## Abbreviations

The following abbreviations are used in this manuscript:

T1D	Type 1 Diabetes
BB-DP	Diabetic-prone rats
BB-DR	Diabetic-resistant rats
MLNs	Mesenteric lymph nodes
EVs	Extracellular vesicles
AHR	Aryl hydrocarbon receptor
ICAM-1	Intercellular adhesion molecule 1
PAMPs	Pathogen-associated molecular patterns
PRR	Pattern recognition receptors
BMDCs	Bone marrow dendritic cells
LPPs	Lipoproteins
LPS	Lipopolysaccharide
Ags	Antigens
PL	Phospholipids
SD	Sprague-Dawley
PBMCs	Peripheral blood mononuclear cells
MACS	Magnetic activated cell sorting
TFs	Transcription factors
Abs	Antibodies
RT	Room temperature
MFI	Median fluorescence intensity
TCR	T cell receptor
PMA	Phorbol-12-myristate-13-acetate
PMA_I	PMA and Ionomycin
CL	Cardiolipin
PG	Phosphatidylglycerol
PE	Phosphatidylethanolamine
DCs	Dendritic cells
PS	Phosphatidylserine
Treg	Regulatory T cells
OAS	2′,5′-oligoadenylate synthetase
VC	Vehicle control
TL	Total lipids
