# Supplementary material for: Lactobacillus johnsonii N6.2 Phospholipids Induce T Cell Anergy upon Cognate Dendritic Cell Interactions
Source: Metabolites. 2025 Apr 22;15(5):284. doi: 10.3390/metabo15050284 (PMC12113001; doi:10.3390/metabo15050284)

Supplementary Materials

Figure S1. Purity and CD3 staining of fresh and cultured T cells.

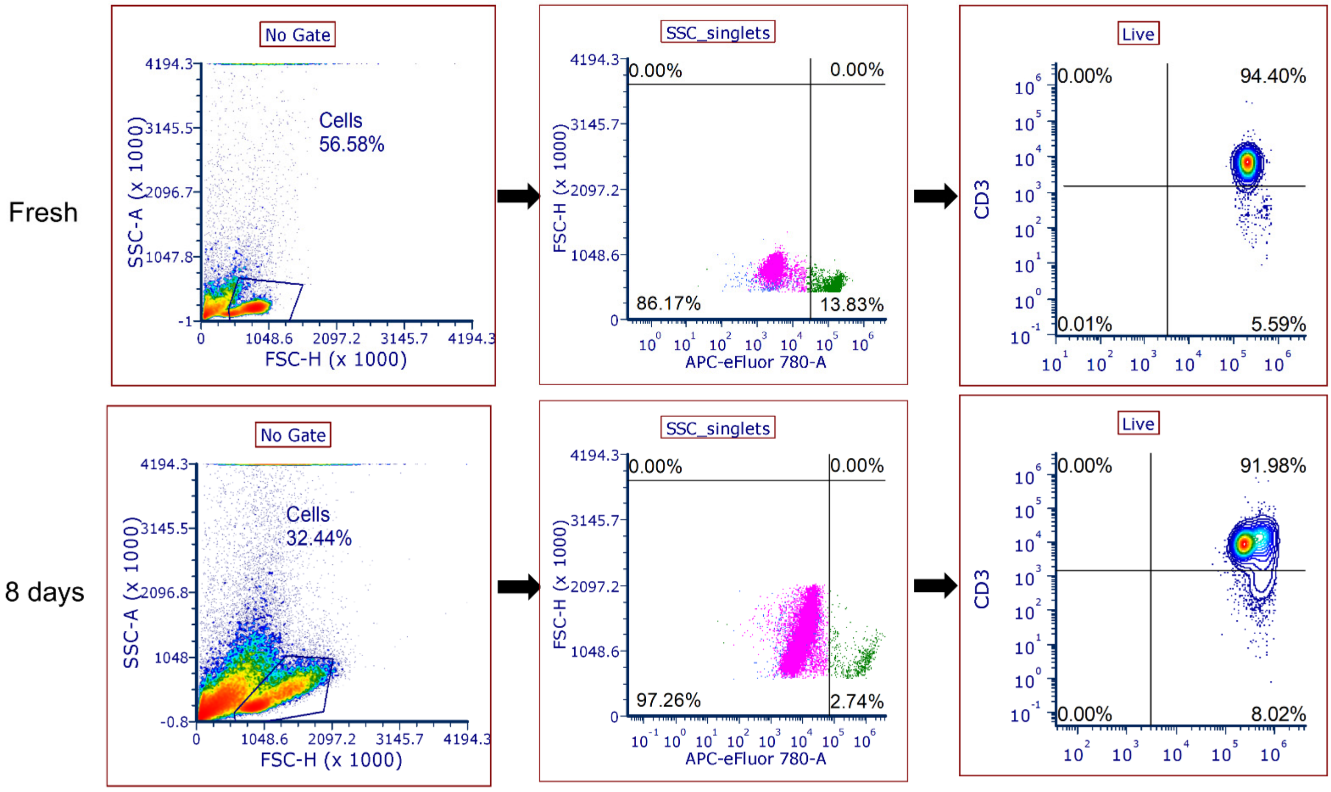

Figure S2. Immunophenotyping of peripheral T cells at the start compared to 8 days of culture

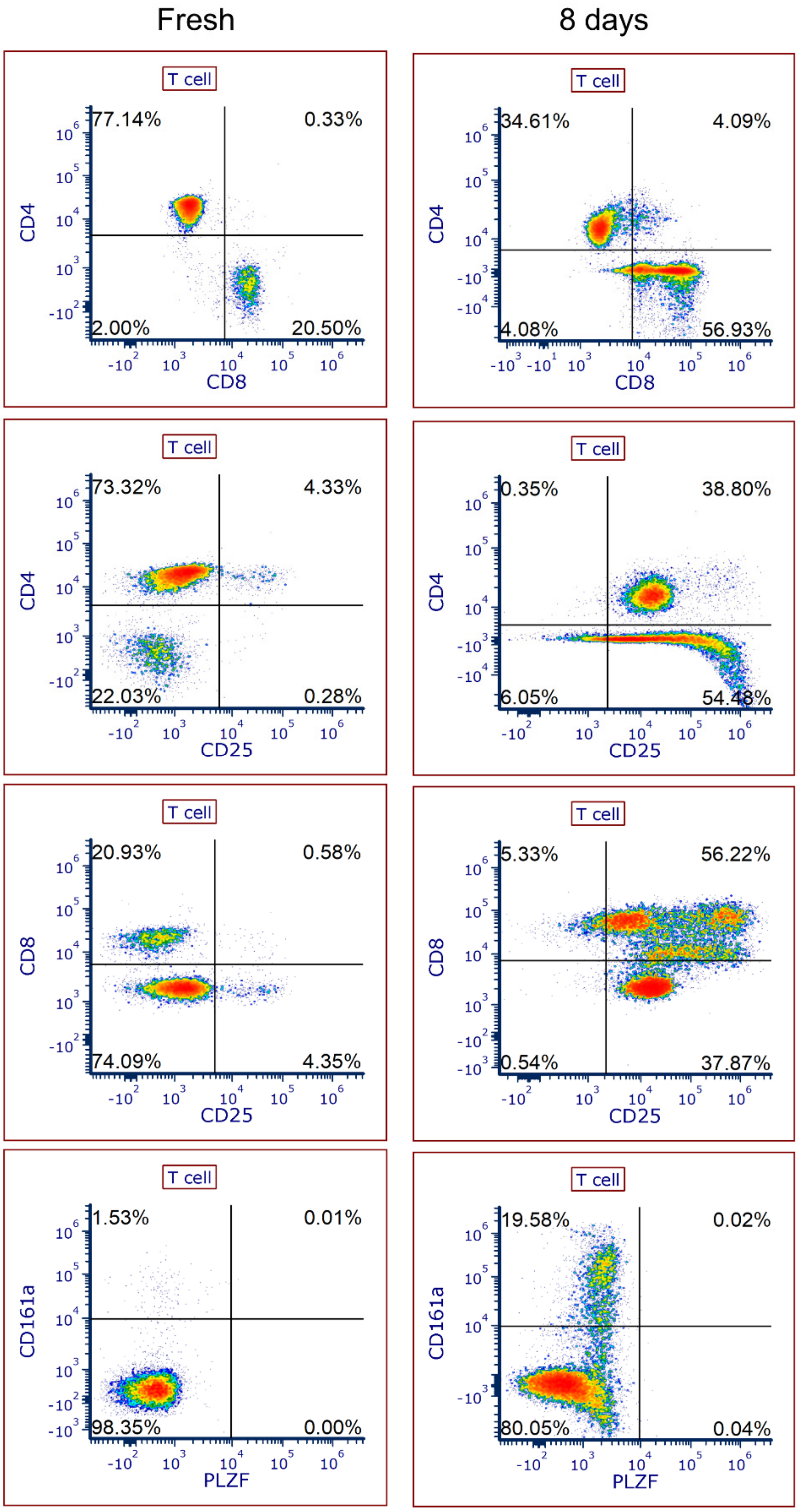

Figure S3. Immunophenotyping of peripheral CD8+ T cells. (a) CD161 staining in CD8+ T cells immediately after isolation compared to those with 8 days of culture. **(b)** FOXP3 expression compared to CD25 and CD161a after 8 days of culture in CD4+ T cells (top left) and CD8+ T cells (other panels).

(a)

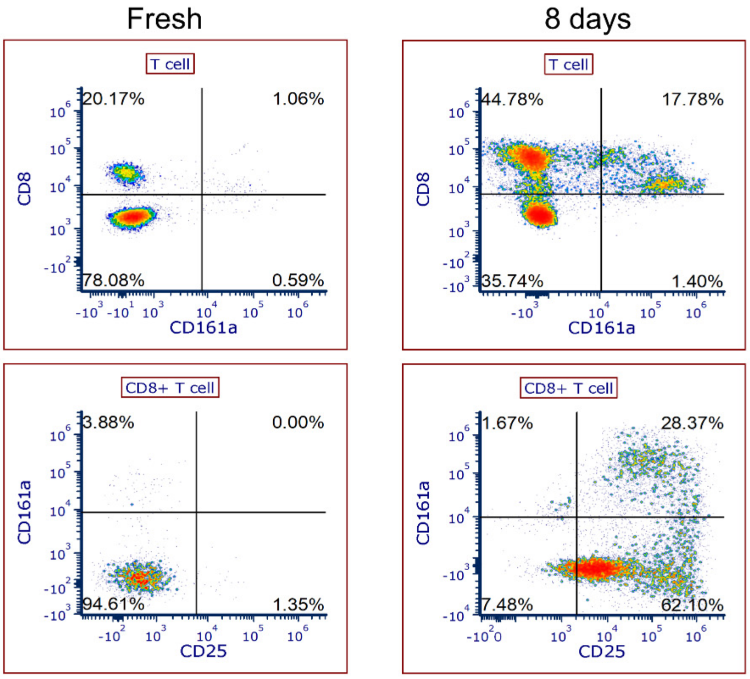

(b)

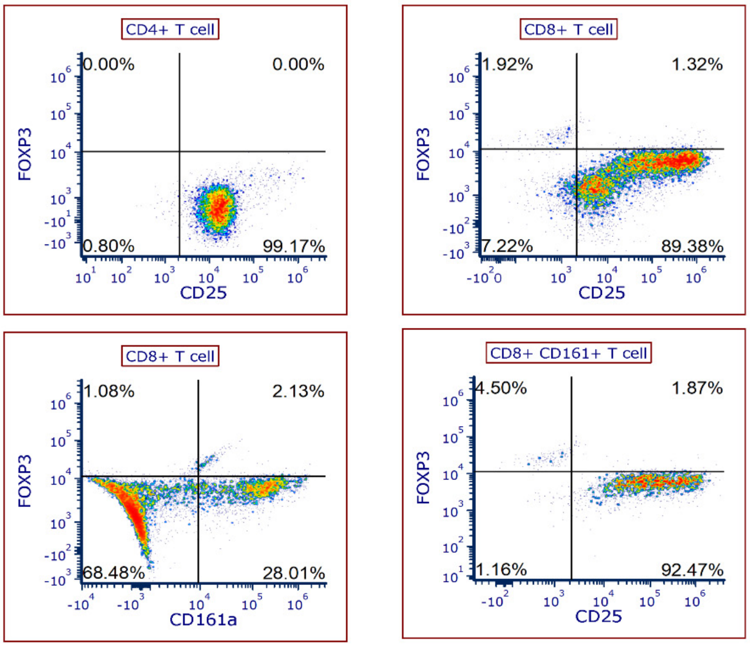

Figure S4. Gene expression levels of Th1 effector-related genes in cryopreserved T cells upon co-culture with PL-stimulated BMDC and TCR stimulation for 18 hours. Gene expression data were normalized to *actb* and results for treatments are presented relative to that of unstimulated T cells. As a positive control, T cells alone were treated with PMA and Ionomycin to induce downstream TCR signaling (PMA_I). Different letter labels denote statistically significant changes (p-value < 0.05). VC: Vehicle control; PL: Phospholipids.

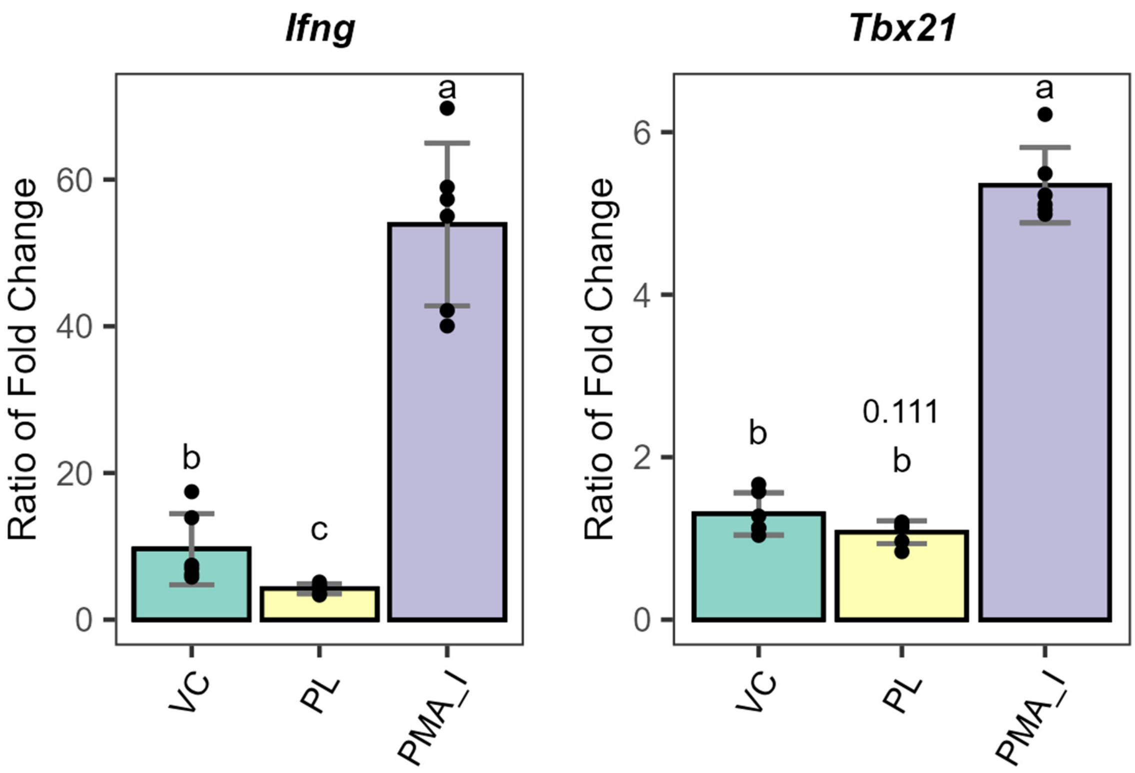

Supplement: Supplementary file 1 [file metabolites-15-00284-s001.zip › Supplementary Material.pdf]
